# Recombination smooths the time signal disrupted by latency in within-host HIV phylogenies

**DOI:** 10.1093/ve/vead032

**Published:** 2023-05-20

**Authors:** Lauren A Castro, Thomas Leitner, Ethan Romero-Severson

**Affiliations:** Information Systems and Modeling, Los Alamos National Laboratory, Los Alamos, NM 87545, USA; Department of Integrative Biology, The University of Texas at Austin, Austin, TX 78712, USA; Theoretical Biology and Biophysics, Los Alamos National Laboratory, Los Alamos, NM 87545, USA; Theoretical Biology and Biophysics, Los Alamos National Laboratory, Los Alamos, NM 87545, USA

**Keywords:** HIV, coalescent, ancestral recombination graph, within-host dynamics, phylodynamics

## Abstract

Within-host Human immunodeficiency virus (HIV) evolution involves several features that may disrupt standard phylogenetic reconstruction. One important feature is reactivation of latently integrated provirus, which has the potential to disrupt the temporal signal, leading to variation in the branch lengths and apparent evolutionary rates in a tree. Yet, real within-host HIV phylogenies tend to show clear, ladder-like trees structured by the time of sampling. Another important feature is recombination, which violates the fundamental assumption that evolutionary history can be represented by a single bifurcating tree. Thus, recombination complicates the within-host HIV dynamic by mixing genomes and creating evolutionary loop structures that cannot be represented in a bifurcating tree. In this paper, we develop a coalescent-based simulator of within-host HIV evolution that includes latency, recombination, and effective population size dynamics that allows us to study the relationship between the true, complex genealogy of within-host HIV evolution, encoded as an ancestral recombination graph (ARG), and the observed phylogenetic tree. To compare our ARG results to the familiar phylogeny format, we calculate the expected bifurcating tree after decomposing the ARG into all unique site trees, their combined distance matrix, and the overall corresponding bifurcating tree. While latency and recombination separately disrupt the phylogenetic signal, remarkably, we find that recombination recovers the temporal signal of within-host HIV evolution caused by latency by mixing fragments of old, latent genomes into the contemporary population. In effect, recombination averages over extant heterogeneity, whether it stems from mixed time signals or population bottlenecks. Furthermore, we establish that the signals of latency and recombination can be observed in phylogenetic trees despite being an incorrect representation of the true evolutionary history. Using an approximate Bayesian computation method, we develop a set of statistical probes to tune our simulation model to nine longitudinally sampled within-host HIV phylogenies. Because ARGs are exceedingly difficult to infer from real HIV data, our simulation system allows investigating effects of latency, recombination, and population size bottlenecks by matching decomposed ARGs to real data as observed in standard phylogenies.

## Introduction

Within-host Human immunodeficiency virus (HIV) evolution impacts between-host HIV evolution on the epidemic level (Volz, Romero-Severson, and Leitner 2017; Leitner 2019). Thus, it is imperative to understand how within-host processes such as recombination and latency shape HIV phylogenies on different levels. Recombination violates the fundamental assumption that every offspring only has one parent (which is fundamental in a bifurcating tree). Instead, recombinant HIV taxa are formed by two parents in a generation, which cause loop structures in the resulting genealogical graph. Depending on the proportion and evolutionary history in each parent, this can lead to errors both in the branch lengths and implied evolutionary relationships among the sampled haplotypes (Mendes, Livera, and Hahn 2019). Latency is the result of HIV provirus in dormant cells, which may be inactive for years, during which no evolution occurs. This may lead to extreme evolutionary rate differences (Immonen and Leitner 2014), causing difficulties in tree reconstruction and especially in time-scaling trees. While these evolutionary effects are well known in the HIV field, they are still often ignored simply because they are difficult to model.

On the epidemic level, the use of pathogen genetic data to infer epidemics has advanced considerably in the past decades. In such analyses, the evolutionary history is nearly always thought of as a bifurcating tree, i.e. a standard phylogeny. Thus, such epidemiological applications (Fisher et al. 2010; Kouyos et al. 2010; Stadler et al. 2013; Volz et al. 2013; Grabowski et al. 2014; Rasmussen, Volz, and Koelle 2014; Ratmann et al. 2016) assume that (1) the genealogy of infection is a primary determinant of the phylogeny and (2) the evolutionary history of a pathogen can be reasonably modeled as a bifurcating process, again, that the sequenced genetic regions are derived from one progenitor. Both assumptions are questionable. Within-host HIV evolution leads to substantial diversity, which has been shown to randomize transmission order and times (Romero-Severson et al. 2014; Giardina et al. 2017; Volz, Romero-Severson, and Leitner 2017; Hall and Colijn 2019). Thus, without modeling the underlying transmission history, the HIV phylogeny will be misleading about the details of transmissions. On a large population scale, where close donors and recipients unlikely are in the limited sample, however, the first assumption may be a reasonable approximation. The second assumption is less understood and may thus present a bigger problem for the application of phylogenetic methods to HIV data.

Recombination is also fundamentally a problem for the epidemiological interpretation of HIV phylogenies, although recombination at the epidemiological level sometimes is more obvious when it involves recombination of distinct so-called subtypes with well-known genetic signatures (Salminen et al. 1995; Siepel et al. 1995). The signal for teasing out recombination events is lower within subtypes and even lower at the within-host level because many infections are established by a single virus (Giorgi et al. 2010; Leitner and Romero-Severson 2018), which limits the extent of parental diversity that recombination detection methods use to identify recombinants. Likewise, the extent of recombination within-host leads to multiple generations of recombination events that further obscure the relationship between genealogy and phylogeny (Song et al. 2018).

Estimates of the rate of HIV recombination within a host are very high. Both empirical (Neher and Leitner 2010) and simulation-based studies (Batorsky et al. 2011) estimate that the effective recombination rate, which incorporates both the probability of coinfection of a single host cell (Josefsson et al. 2013) and the template switching rate, is on the order of 1.4 × 10^−5^ to 1.38 × 10^−4^ per base per generation, comparable to the estimated point mutation rate of 2.2−5.4 × 10^−5^ per base per generation (Mansky and Temin 1995; Gao et al. 2004). Within a host, recombination provides HIV-1 with a means to increase the genetic variation for selection to operate on (Fisher 1930; Muller 1932; Crow and Kimura 1965; Felsenstein 1974; Michod, Bernstein, and Nedelcu 2008), can lead to rapid emergence of antiviral drug resistance (Gu et al. 1995; Kellam and Larder 1995; Moutouh, Corbeil, and Richman 1996), and can help shed deleterious mutations. Theoretical studies have shown that the magnitude of benefits brought by recombination depends on the interaction between factors such as population size and epistatic interactions (Kondrashov and Kondrashov 2001; Michod, Bernstein, and Nedelcu 2008; Moradigaravand et al. 2014).

Recombination clearly plays a central role in the evolutionary process of within-host HIV evolution, yet there are very few methods for modeling and addressing recombination empirically. Inference of more generalized recombination graphs called ancestral recombination graphs (ARGs) has been proposed and demonstrated (Hein 1990; Griffiths and Marjoram 1996; Rasmussen et al. 2014), but those methods are not scalable to large trees (Wang, Zhang, and Zhang 2001). Even if robust methods for inferring ARGs from data existed, there is very little understanding of how to interpret ARGs or how those results could be used to understand the interplay between recombination and other evolutionary processes. In this paper, we develop a coalescent-based simulation method for simulating ARGs under a range of conditions, including the rate of diversification, the level of recombination, the extent of latency, as well as within-host population bottlenecks in an idealized population intended to represent within-host HIV evolution. We implement a decomposition method to map ARGs simulated under a variety of conditions onto bifurcating trees. This method allows us to investigate the effects of within-host HIV biological processes on familiar phylogenetic trees without having to make unrealistic assumptions about the underlying genealogy. Combining the simulator with approximate Bayesian computation (ABC) methods and genetic data from nine serially sampled HIV patients, we also look at levels of recombination, latency, and population dynamics that are consistent with the real-world within-host HIV phylogeny.

## Methods

### Methodological overview

We simulate ARGs using a coalescent method that begins with a set of given samples at various times post-infection and simulates an ARG backward in time according to a set of parameters intended to model the effects of recombination, latency, and selection. To map the simulated ARGs into the space of bifurcating trees (i.e. what we can infer using standard phylogenetic methods), we decompose each ARG into a bifurcating tree for each residue in simulated sequences by assuming a single random break point delineating the contribution from each parent. The average distance between tips is then computed over the population of decomposed trees and used to reconstruct a single tree that averages over the set of unobserved recombination events. Our ABC approach is based on comparing empirically measured statistics from real within-host HIV data to statistics computed on the simulated decomposed trees.

### Model assumptions

We assume that an individual is infected with a single transmitted HIV variant. From that lineage, the HIV population diverges and diversifies linearly with time, with intermittent demographic bottlenecks caused by the host immune response. In addition, we assume that there is a reservoir latent population that is established 3 weeks after infection. The latent reservoir is assumed to be constant in size such that all movement to and from the latent reservoir is balanced; we call this the global flow rate. Integrated provirus in the latent reservoir does not acquire new mutations. For all processes, we assume neutrality, specifically that any lineage is equally likely to coalesce, recombine, go into or out of the latent reservoir, or survive a population bottleneck event. Finally, we assume that the evolutionary rate remains constant over the course of the infection.

### Simulation of the ARG

We simulate ARGs in reverse time, beginning with the fixed set of sample times and moving backward in time until the time of infection. All lineages have a state of being either *latent, L*, or *active, A*, and at any point in time, there are *$k_L$* latent lineages and $k_A$ active lineages. We model four possible events: (1) a recombination event between two active lineages, (2) a virus entering the latent reservoir, (3) a virus in the latent reservoir reactivating, and (4) a coalescent event between two active lineages. We assume that waiting times to each event are independent and conditional only on the extant number of lineages in either an active or a latent state and the time since transmission. The model uses the parameters listed in Table 1.

**Table 1.** Parameter table.

Symbol	Description	Ranges	Citations
$\alpha $	Number of transmitted lineages	0	
$\beta $	Population growth rate	1	
$\rho $	Rate of recombination per lineage	{0, 0.001, 0.01, 0.05, 0.1}	Neher and Leitner (2010); Batorsky et al. (2011); Jetzt et al. (2000); Zhuang et al. (2002)
$\lambda $	Global flow rate of a lineage into and out of the latent reservoir	[0.5–5.25]^*^	Siliciano et al. (2003)
*N_L_*	Size of the latent pool	[10^1^–10^3^]	Siliciano et al. (2003); Finzi et al. (1997); Chun et al. (1997); Wong et al. (1997); Ho et al. (2013)
B_strength_	Population size (*N*) during demographic bottlenecks	[1–100]	
B_frequency_	Frequency of bottlenecks (days)	[14–365]	
*k_A_*	Number of active lineages		
*k_L_*	Number of latent lineages		

*This range of λ corresponds to these $N_L$ values using the equation $\lambda=N_L\frac{log(2)}{44x30}$.

### Simulation events

#### Coalescence

When a coalescent event occurs, two extant active lineages are chosen randomly and joined to form a single lineage (2*A* → *A*), and a coalescence node is inserted into the ARG. The expected waiting time for two random lineages to coalesce is dependent on the effective population size and the number of active lineages. The population size is modeled as $N\left( t \right) = \alpha + \beta t$, where $\alpha $ is the number of transmitted lineages, $\beta $ is the growth rate per generation (assumed to be 1 generation per day), and *t* is the time since infection in days. The linear growth model is motivated by empirical observations of diversity trends (Shankarappa et al. 1999; Zanini et al. 2015), as previously implemented (Romero-Severson et al. 2014; Romero-Severson, Bulla, and Leitner 2016). For all simulations, we assume that $\alpha = 0$ and that $\beta = 1$. $\alpha $ is set to 0 to ensure that all lineages coalesce by the time of infection. During bottleneck events, the population remains at a constant size, ${B_{{\rm{strength}}}}$, for a period of 5 days.

Thus, population growth can be described by the following system of equations:


$$N\left( t \right) = \left\{ {\begin{array}{*{20}{c}}
{\alpha \, + \,\beta t,\quad\,\,\, \rm{mod}\left( \rm{t,\,{B_{{\rm{frequency}}}}} \right) \ge \rm5}\\
{{B_{{\rm{strength}}}},\quad \rm{mod}\left( \rm{t,\,{B_{{\rm{frequency}}}}} \right) \lt \rm5}
\end{array}} \right.$$

Following the period of decreased population size, the population resumes linear growth, maintaining the overall gradual linear increase in genetic diversity over the course of the infection. In this way, we approximate the effects of selection without explicitly incorporating the reproduction probability of individual lineages. In the normal regime, the time to the next coalescent event is computed in the way described by Romero-Severson, Meadors, and Volz (2014).

During periods of bottlenecks that occur every ${B_{{\rm{frequency}}}} = 300$ days, the time to the next coalescent event is an exponential random variable with rate $\left( {\frac{{{k_A}\left( {{k_A}\, - \,1} \right)}}{{2N}}} \right)$ (Wakeley 2009) where $N = {B_{{\rm{strength}}}}$. By chance, the parameters of $B_{\mathrm s\mathrm t\mathrm r\mathrm e\mathrm n\mathrm g\mathrm t\mathrm h}$ and ${B_{{\rm{frequency}}}}$ could occur such that $N = {B_{{\rm{strength}}}}\, \gt \,N\left( t \right)$. Using *t *= 14 as the earliest time a bottleneck could occur, we found this phenomenon to occur in 0.88 per cent of all bottleneck events in our dataset. As these bottlenecks represented a small fraction of the total number of events within affected simulations (Figure S1), we determined the effect to be minimal. The Kingman coalescent is generally considered to be valid when the sample size is much smaller than the population size. However, as previously demonstrated (Romero-Severson, Bulla, and Leitner 2016), the error caused by large sample sizes in the linear coalescent is relatively small compared to the Kingman coalescent.

#### Recombination

A recombination event adds a lineage to the total number of extant active lineages (*A* → 2*A*). When a recombination event occurs, one lineage is chosen from the set of extant active lineages and is split into two lineages representing each parent. A node representing the recombination event is inserted into the ARG, and a single break point is assigned to that node with uniform probability over the set of simulated residues. We assume recombination to be a homogeneous process where recombination events occur at rate $\rho $ per lineage, and thus, the time to the next recombination event is an exponential random variable with rate $\rho {k_A}$.

#### Latent reservoir deposition and reactivation

The size of the latent reservoir, *N_L_*, is assumed to be constant with a fixed half-life of 44 months (Siliciano et al. 2003). Therefore, the per day rate of lineages moving into and out of the latent reservoir is $\lambda = {N_{L\,}}\frac{{log\left( 2 \right)}}{{44x30}}$; i.e. a larger latent reservoir corresponds to a larger global flow rate into and out of the reservoir to maintain a constant half-life.

The overall rate of activation and deposition is equal, but we also need to consider the probability that an activation/deposition event is ancestral to the sample. For activation (*L* → *A*), the time to the next event is an exponential random variable with rate $\lambda \frac{{{k_L}}}{{{N_L}}}$. For deposition (*A* → *L*), the time to the next event is given by the same equation that governs the time to the next coalescent event with the $\left( {\frac{{{k_A}}}{2}} \right)$ replaced by $\lambda {k_A}$. Both deposition and reactivation events are recorded in the ARG as a node along a single branch indicating that the state of that lineage has switched.

#### Longitudinal sampling

To simulate longitudinal sampling, we designate times along the reverse time axis when new active lineages are added to the simulation. At each additional sampling event, the branch lengths of any remaining active and latent lineages from the previous sample are extended in time up to the next sampling time. From there, the simulation proceeds as before, with ${k_A}$ updated to reflect the additional new samples. The simulation ends after the last sampling time (first in forward time) when ${k_A} = 1$ before $t = 0$.

### Mapping the ARG to a single bifurcating tree

**Figure 1.**
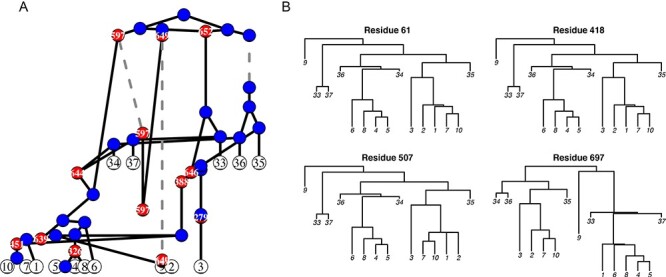
ARG decomposition into a series of bifurcating trees. (A) The simulated ARG from two sampling events. Red dots represent recombination events, with the break point identified in the white text. Blue dots represent coalescent events between two lineages. Dashed gray lines represent when a lineage is in the latent reservoir. Branch lengths correspond to time. This ARG represents the genealogical relationships among five viruses sampled 3.5 months post-infection and ten viruses sampled 7 months post-infection and was simulated using *ρ* = 0.08, *N_L_* = 760 assuming a sequence length of 1000. (B) Each panel shows the decomposition of the ARG into a bifurcating tree for a specific position in the alignment. Recombinant (red) nodes are resolved at the nucleotide level by removing one of the parent branches depending on where the recombination event occurred with respect to the given alignment position. If no recombination occurred between two positions, then the genealogy for those positions will be identical (e.g. Residues 61 and 418). However, recombination can alter both the topology and branch lengths of the underlying genealogy (e.g. Residues 507 and 697).

**Figure 2.**
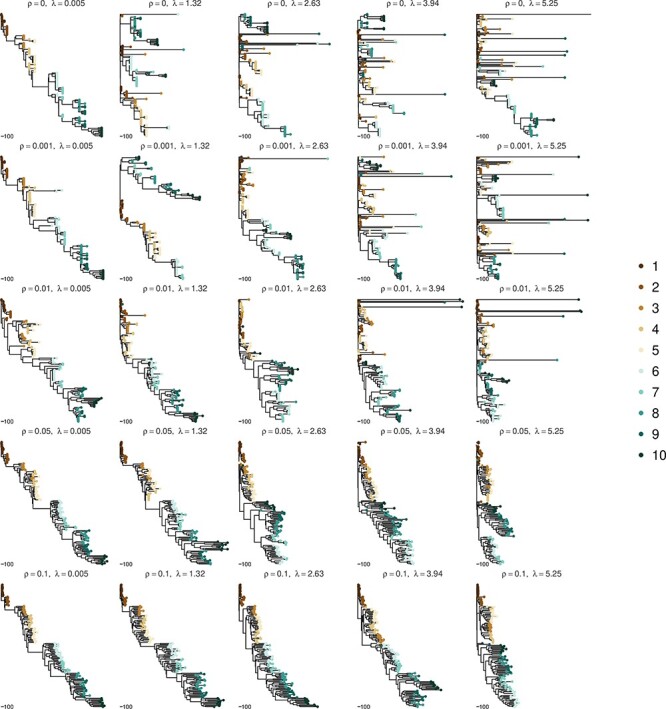
Simulated trees under a range of recombination rates and global flow rates in and out of the latent reservoir. This figure shows a simulated within-host phylogeny under the specified recombination rate, *ρ*, and the global flow rate, *λ*. The global flow rate increases from left to right (more frequent deposition and activation of latent lineages) and the recombination rate increases from top to bottom. The color of the tips represents the time from infection in years. Trees are all on the same scale (days).

To map the simulated ARG into what we might observe with standard phylogenetic methods, we (1) decompose the ARG into a population of bifurcating trees, one for each residue in the simulated sequence (Fig. 1), (2) compute an average distance matrix from the population of bifurcating trees, and (3) compute a single phylogeny from the average distance matrix. For each residue, *i*, in the simulated sequence, we extract a single bifurcating tree by removing recombination nodes by trimming the right-hand lineage if *i ≤* *y* where *y* is the break point at that node and trimming the left-hand lineage otherwise. For each bifurcating tree, we compute the distance between each tip by traversing the tree using the igraph package (Csardi 2019) in R and summing the total time along that path that was in the active state. During the latent state, no evolutionary time is accumulated (i.e. we assume that there is no potential for mutation in the proviral state). Finally, we use a minimum evolution principle (Desper and Gascuel 2002) (similar to the well-known neighbor joining method) to generate a hierarchical clustering representation of the average distance matrix. To visualize a hierarchical clustered tree, we root it at the most recent common ancestor of the samples from the first (in forward time) sampling event. Hereafter, we refer to this as the *simulated tree*. Because of computational limitations, we complete this decomposition process for a random sample of 400 residues out of the 700 simulated. As the number of random sampled sites approaches half, we observe that the hierarchical clustering representation is similar to one generated using all 700 sites (Figure S2).

### Data

We applied this framework to the analysis of longitudinal HIV-1 deoxyribonucleic acid sequences sets from nine patients (Shankarappa et al. 1999). In that study, sequences corresponding to the HIV-1 *env* gene were taken from each participant over a course of 6–12 years starting at 3 months from the time of seroconversion. There was an average of 11.875 individual sampling events with an average of 9.83 (SD 1.66) samples taken per event. We aligned the sequences of each participant using MAFFT v7.305b2 (Katoh and Standley 2013) and generated a tree using a standard maximum likelihood method, PhyML v3.1 (Guindon et al. 2005), under a GTR + G + I substitution model with Nearest Neighbor Interchange and Subtree Prune and Regraft  search. For each patient, we simulate a population of ARGs under a range of different parameter values and decompose those ARGs into a single tree as described earlier. Therefore, for each patient, we have 31,100 simulated that we use for inference based on our set of statistics. To compare within-host evolutionary dynamics over the course of the same time duration for each patient, we restricted our analysis to samples taken no more than 90 months past the time of seroconversion.

### Selected statistics, objective function, and parameter inference

Our inference method is an ABC method using importance sampling to generate a population of parameters that produce simulated data that are most similar to the empirical data with respect to a set of statistics. To capture the general aspects of the empirical phylogenies, we used five statistics (Table 2): the Sackin index (SI), the external to internal branch length ratio (EI ratio), the mean number of lineages through time (MLT), the coefficient of variation (CV), and the relative pairwise difference (RPD). SI and EI are computed using standard methods. The MLT is a proxy measure of phylogenetic temporal structure in a longitudinal set of samples, defined as the mean number of branches linking two consecutive samples. For example, if all the tips from a sample coalesce with one another, then the number of lineages through time for that sample is one. On the other extreme, if each tip from a given time point co-clusters with a tip from another time point, then the number of lineages through time is equal to the number of tips. The CV is the mean of the ratio of the SD of all pairwise distances in a given time point to the mean of those distances computed over all sampling time points. When the CV is low, the distribution of branch lengths has low heterogeneity (i.e. all branch lengths in a given time are very similar). The RPB is the element-wise difference in the full cophenetic distance matrix for the simulated and empirical trees. To make the matrices directly comparable, we compute differences in the simulated and empirical matrices within- and between-sample times of the same rank, e.g. for sample time one we compute the absolute sum of the ordered distances within that time point and so on for all combinations of time points. Because the simulated and empirical distance matrices are on different scales (time and generic distance, respectively), we further assume that they are related by an unknown multiplicative scalar quantity. Rather than trying to fix that scalar, we optimize it for each comparison to minimize the element-wise difference in the simulated and empirical distance matrices. This allows us to directly compare the relative differences in the simulated and empirical distance matrices without worrying about the effect of the unknown scaling factor at the price of losing the ability to distinguish between very similar trees at different scales.

**Table 2.** Statistical probes used to match the simulated evolutionary history to a patient’s empirical HIV-1 *env* phylogeny.

Statistic	Type	Description
SI	Topological	The average number of splits from a tip to the root of a tree and captures asymmetry over the evolutionary history of the sample
MLT (Giardina et al. 2017; Janzen, Höhna, and Etienne 2015)	Time structure	The diversification of lineages normalized by the number of extant tips
EI ratio	Distance	The ratio between the mean external branch length (branch that ends with a sampling event) and the mean internal branch length (branch between coalescence events)
RPD	Distance	The difference in the empirical and simulated distributions of branch lengths ranked by size within each sampling event
CV in pairwise distance	Distance	The ratio between the standard deviation of pairwise distances between branches of the same sampling event and the mean pairwise distance between branches of the same sampling event

*Notes*: Some statistics measure tree characteristics (topological or time structure), while others measure distance statistics (distance). A simulation’s score, *d*, is the sum of the normalized differences between the statistic measured on reconstructed simulated tree and the empirical tree.

The objective function (i.e. the metric defined on the space of sample statistics) is then defined as the sum of the absolute difference in each normalized statistic. If the objective function is 0, then the simulation has the exact same values of the measured statistics as the empirical tree for a given patient. To search for parameter sets that produced trees that are most similar to the empirical trees, we drew 31,100 parameter sets within fixed strata of the recombination rate. Within each recombination, strata parameters were sampled from [0.5−5.25] for $\lambda $, [1−100] for ${B_{strength}}$, and [14−365] for ${B_{frequency}}$, with uniform probability and assuming independence. We used the top 5 per cent (i.e. with the lowest score) of parameters as the posterior distribution of parameters for each patient.

## Results

### HIV recombination and latency interact and affect the observed temporal signal in a within-host HIV

#### Phylogeny

To qualitatively investigate the interplay between recombination and latency, we first simulated a single ARG for a range of recombination rates and global flow rates in and out of the latent reservoir of infected cells and reconstructed the corresponding bifurcating phylogeny (Fig. 2). Each tree has the same sampling scheme covering 132 samples over 147 months, which is representative of a densely sampled within-host HIV phylogeny. The top left tree represents what happens when there is a very small effect of latency and no recombination. In this panel, we see a tree that is orderly with respect to time (i.e. the tips sampled at the same time, more-or-less, occur at the same tree height). Moving along the top row (no recombination) from left to right, the latency rate increases, and we see almost immediately clear signs of temporal disordering: (1) large sets of sequences sampled at later times mixed with early samples, (2) lineages that appear to be extremely divergent from other sequences sampled at the same time, and (3) a collapse of the ladder-like backbone of the tree structure. Pattern 1 occurs when a period of latency happened on a branch that is ancestral to several tips that are sampled later, while Pattern 2 occurs when little or no latency occurs on a single branch leading to an apparently highly divergent taxon. Pattern 3 is a result from randomly inserting ancestral variants from the latent reservoir, which destroys the basal time structure. Thus, in the absence of recombination, latency can completely disrupt the temporal signal in a phylogeny.

In the left column, moving from top to bottom, the recombination rate is increased and the level of latency reactivation is low. In general, even high levels of recombination do not disrupt the temporal signal; however, it can cause some co-clustering of samples from different time points, but still overall has a distinct ladder-like structure. Interestingly, the higher levels of recombination in this present study tended to produce longer external branch lengths, which could easily be confused as a signal for exponential growth. In general, moving from top to bottom, at all levels of latency tested, recombination clearly ‘recovers’ the temporal signal inherent in the data even at very high levels of recombination. At recombination rates above 0.05, we no longer see highly divergent lineages as we are only sampling recombinants of those lineages with other less diverged lineages (i.e. those with periods of latency in their past). However, when both the recombination and latency are high, it is still possible to see a slight disordering of the temporal signal as later sampled tips can co-cluster with earlier samples.

### Combined evolutionary effects generate the familiar HIV within-host phylogenetic structure

Our coalescent ARG simulator included several evolutionary processes that each could violate the fundamental assumptions on which standard phylogenies are based. As we have already seen in Fig. 2, recombination rescues the disruptive effects of latency. This is surprising because recombination itself severely violates the bifurcation assumption. Thus, we next investigate this theme of recombination apparently amplifying, or at least rescuing, the phylogenetic signal disrupted by other evolutionary processes, also including periodic population bottlenecks. These periodic bottlenecks were introduced to mimic population size effects due to selection and other demographic processes, but the overall model is still a neutral model that cannot capture adaptive and selection-driven genetic effects.

**Figure 3.**
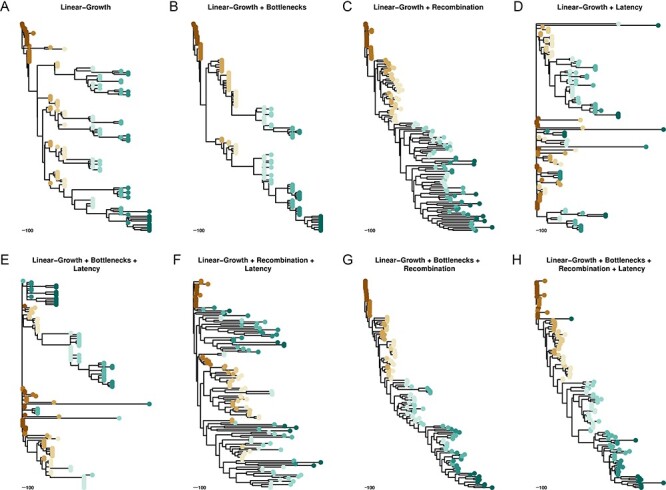
Illustration of effects of within-host evolutionary processes on virus phylogenies. Each tree is the decomposed average tree from one ARG simulation of 12 years using 132 sampled taxa spread over ten sampling events. Color represents tips sampled at the same time. The baseline model, a linear-growth coalescent model is in Panel A. Panels B-H illustrate the effect of the processes listed at the top of each panel. For simulations in which the process is active: ${B_{{\rm{frequency}}}} = 300$ and ${B_{{\rm{Strength}}}} = 15$; *λ* = 1.31; and *ρ *= 0.01. Trees are all on the same scale (days).

Real within-host HIV phylogenies based on sequences sampled over time characteristically display unbalanced, ladder-like, time-ordered trees (Shankarappa et al. 1999; Zanini et al. 2015). With more frequent sampling over time, lineages from different samples will overlap more, and conversely, over longer time, only a few lineages survive from one sampling time to the next (Immonen et al. 2015). Simulating actual ARGs that include population demographics, recombination, and latency makes it possible for us to investigate such effects on the expected bifurcating phylogeny that we are accustomed to see in HIV evolutionary research. We simulated within-host evolution over a 12-year period, sampled roughly yearly, to illustrate the effects that complex evolutionary processes have qualitatively on bifurcating trees. Overall, each of the evolutionary processes we simulate in our ARG framework shows clear qualitative effects on the decomposed bifurcating trees (Fig. 3).

Figure 3A shows an example of a tree generated using only a linear growth coalescent model that accounts for linear increase in genetic diversity from the time of infection (Romero-Severson, Meadors, and Volz 2014). This model captures the lengthening of external branches as the infection progresses that is observed in empirical within-host HIV phylogenies. However, the long-term co-existence of distinct clades produced by simple linear growth coalescent models is inconsistent with reality. While some HIV-infected patients’ HIV populations can show multiple lineages surviving over time (Skar et al. 2011), this tree has too many parallel lineages co-existing and diversifying to appear fully realistic. As expected, adding bottlenecks to the simulation cuts down on the number of co-existing lineages (Fig. 3B) as not all co-existing lineages will survive a bottleneck event. Surprisingly, adding only recombination to the linear growth produces trees that look plausibly like within-host HIV phylogenies (Fig. 3C). This is due to the fact that recombination has the effect of averaging over extant heterogeneity (i.e. co-existing lineages recombine with each other and remove the more diverse parents), which produces trees that look like they have the signal of weak selection but, as stated earlier, do not actually simulate real selection, only the population size effects associated with selection. Without either population bottlenecks or recombination to trim and integrate the re-emergence of latent viruses (Fig. 3D), phylogenies simulated with linear growth and latency alone do not resemble within-host HIV phylogenies at all. With latency in the model, adding bottlenecks alone cannot recover the expected tree shape (Fig. 3E), while recombination can (Fig. 3F). The tree in Fig. 3F shows the characteristic co-clustering of heterochronous samples that is expected of a long period of evolutionary latency, but the branches appear very long suggesting long periods of population growth uninterrupted by selection. The final two trees (Fig. 3G and H) show how these evolutionary effects, working simultaneously, can produce HIV phylogenies that very closely resemble real phylogenetic trees observed from longitudinally sampled patients.

### Phylogenetic probes in bifurcating trees respond to recombination and latency activity

**Figure 4.**
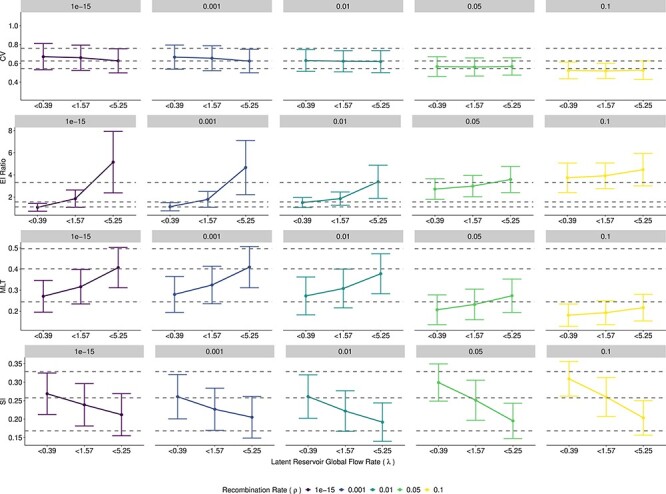
The magnitude of each biological process causes variability in distance and topological statistics. Each dot represents the mean and SD of the statistic. Colors represent different rates of recombination, *ρ*, with recombination values increasing from left to right. Simulations are grouped into three levels of the global flow rate, *λ*, of increasing magnitude, with the break points depicted on the *x*-axis. The gray dashed lines indicate the maximum, median, and minimum value for the nine empirical patients in the study by Shankarappa et al. (1999). On the *y*-axis, the absolute limits of the MLT and the SI are from (0, 1). We have shown that both the empirical and simulation values are a subset of this possible range.

**Figure 5.**
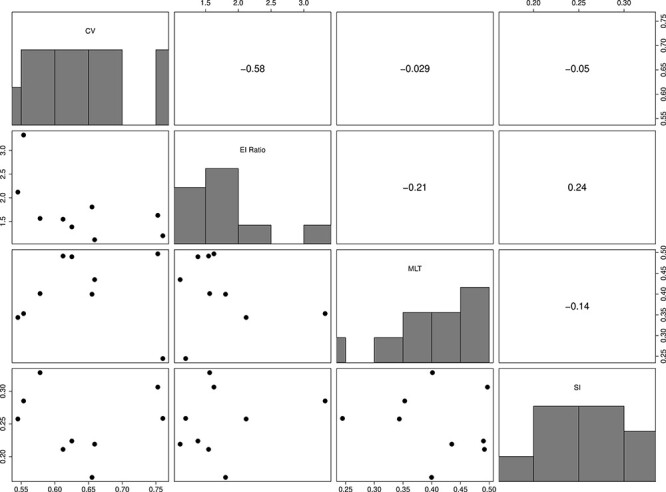
Tree statistic probe values for the nine patients. These statistics were used to match simulations to the patient observed HIV-1 phylogenies. The lower left scatter plots depict the values of each corresponding row and column metrics for each patient. The diagonal is a histogram of the statistic values across the nine patients. The upper right triangle gives the correlation of the corresponding row and column metrics. The CV captures the relative variability in the pairwise distance of tips from the same sampling event, the EI ratio describes the relative differences between external and internal branches, the MLT calculates the mean number lineages of lineages in a clade with extant descendants across sample times, and the SI is an indicator of tree balance. We used an additional statistic, the RPD; however, we do not depict it here as it is a relational metric calculated directly between a simulated distance matrix and the observed empirical distance matrix.

Next, we investigated effects on the statistics used to match simulated and real HIV phylogenies (Fig. 4). All the statistics are variable under a broad range of both the recombination rates and global flow rates in and out of the latent reservoir. That is, these statistics clearly respond to both of these important biological processes suggesting that, collectively, they form a reasonable probe for an ABC-based inference and matching of simulated to real phylogenies. The empirical distributions of the statistics for nine HIV-1-infected patients are shown in Fig. 5.

All the selected statistics show high levels of heterogeneity between patients, possibly suggesting variability in both recombination and latency levels between patients. However, Fig. 4 also shows that stochastic effects produce fairly wide distributions in the statistics, which could also explain some of the between-patient heterogeneity in the within-host phylogenies. Interestingly, higher recombination rates reduce the variance in all the statistics, which is most pronounced in the EI ratio. This is expected because recombination, in general, averages out differences between contemporary viruses at the sequence level and therefore should reduce stochastic effects between simulation runs. Overall, our method of simulating ARGs, decomposing them into bifurcating trees, reconstructing a consensus tree, and measuring the tree statistics on those trees is valid in that it produces self-consistent results that are linked to biological processes through our ARG simulator.

### Evaluation of within-host heterogeneity among real HIV-1-infected patients

**Figure 6.**
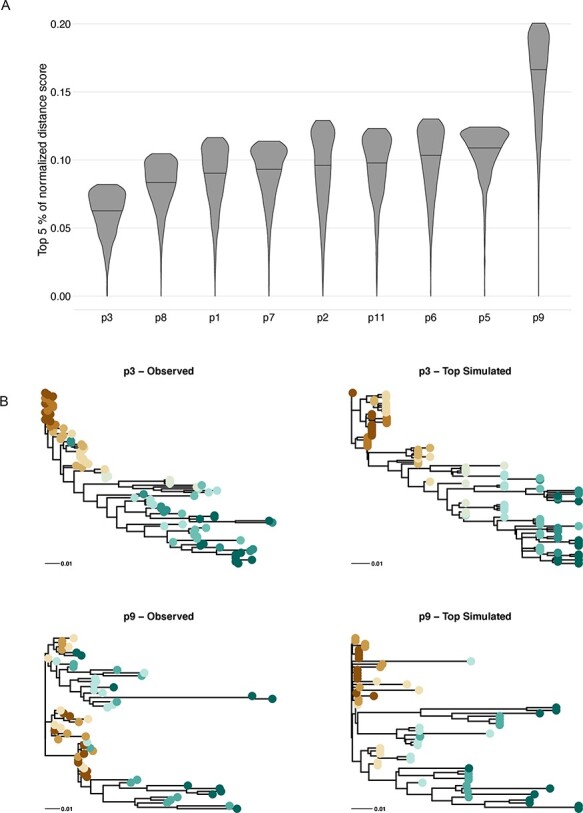
Normalized distance scores of the best-fitting 5 per cent of simulations for each patient. Panel (A) shows the density of the best-fitting 5 per cent of 31,100 distance scores normalized for each patient, with the best-fitting match receiving a score of 0 and the worst-fitting match receiving a score of 1. Patients are ordered by the mean of the normalized top scores. Panel (B) illustrates the variation in quality of model fits by showing the observed and single best simulation for Patients 3 and 9. The simulated trees are scaled from time to genetic distance by the optimal evolutionary rate found when calculating the RPD.

While this paper was focused on studying qualitative effects of combined latency, recombination, and population bottlenecks on a typical HIV phylogeny, we also evaluated different levels of each of these evolutionary processes, including when matching simulations to real within-host phylogenies. Thus, we could estimate how our model parameters varied across patients. Overall, the simulator was able to produce trees that resembled real patient data by minimizing the ABC distance between the observed and simulated tree statistics (Figure S3). Figure 6 (Panel B) shows two examples of empirical and matched simulated trees. The simulated tree with the best score for Patient 3 shows several of the typical tree statistics of the empirical tree, including samples from specific time points at similar height and the general ladder-like tree structure with increasing external branch lengths in later time samples. While the simulator worked well for most of the patients, some of the patients showed overall lower scores in the posterior samples (Fig. 6, Panel A) suggesting that their results are not as reliable (e.g. p9). HIV evolution is often patient specific (Lee et al. 2008; Immonen et al. 2015), in part due to unique immune pressures that we do not model here. Thus, for comparison, we evaluated trees computed on third codon positions only to assess possible effects of selection on our statistics (Figure S4). Third codon site–based trees have less mutational information, making them less robust (approximate likelihood-ratio test node support values significantly decreased, *P* = 1.5e-8–6.2e-3, Wilcoxon signed-rank test), and are therefore less reliable. As expected, because there are fewer mutations, third codon site–based trees showed somewhat higher EI ratios, as well as higher number of lineages through time, while SI showed no systematic difference, i.e. it was sometimes a little higher and other times a little lower than the full data trees, again explained by the increased uncertainty when using fewer sites.

**Figure 7.**
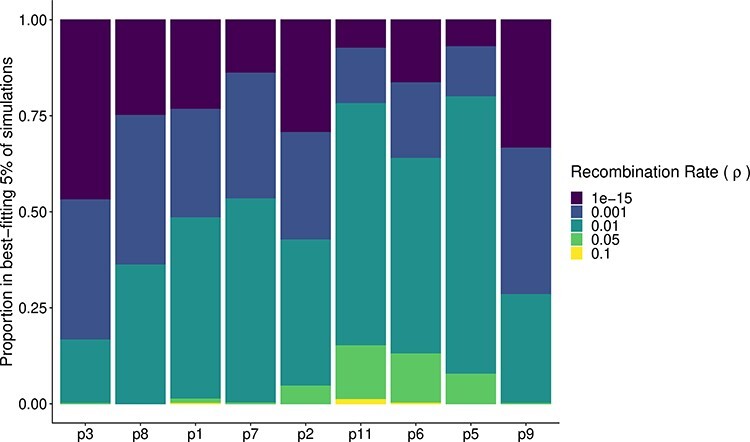
The recombination breakdown within the best-fitting 5 per cent of simulations for each patient. For this figure, we bootstrapped the scores within each recombination strata so that each stratum contained 10,000 values. For each bootstrap replicate, we normalized the score across the 50,000 samples and calculated the relative frequency of each recombination rate in the top 5 per cent of matching simulations. Here, we plot the mean relative frequency of each recombination strata over 100 bootstrap replicates. Patients are organized by the mean of the normalized top scores, with Patient 3 having the lowest (best-fitting) mean score.

**Figure 8.**
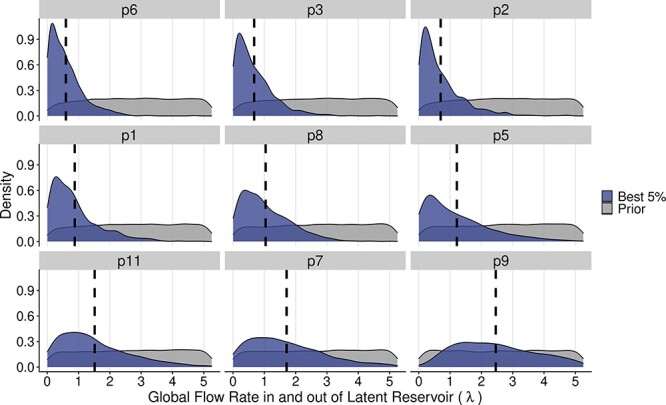
Marginal distributions of the global flow rates in and out of the latent reservoir across the nine patients. Colors distinguish the density of parameters between simulations with the best-fitting 5 per cent of distance scores (blue) and the highest 95 per cent of distance scores (gray). The dashed line indicates the mean of λ for the best 5 per cent distribution. Patients are ordered by increasing values of λ of the best-fitting 5 per cent of simulations. This figure represents the results of one of the bootstrap replicates.

The signal of recombination in the posterior samples for each patient is shown in Fig. 7. For six of the nine patients, the recombination value of 0.01 recombination events per lineage per generation represented the plurality of recombination values in the posterior (excluding p3, p8, and p9). Across patients, 0.01 represents an average of 45 per cent of the posterior, and the second most common recombination value of 0.001 represented on average 27 per cent of the posterior. Overall, this implies recombination rates that are substantially lower than previously estimated values. On the other hand, the global in and out of latency rate implied a size of the latent reservoir that was generally well constrained by the data and showed only modest levels of heterogeneity between patients (Fig. 8). Excluding Patient 9, the mean global flow rates were in the lower half of the tested range, suggesting only a relatively small number of latency reactivation events were required to explain the empirical phylogenies. Interestingly, there is a positive correlation in the recombination rate and the global flow rate in and out of the latent reservoir (Figure S5), suggesting that if the latent reservoir is very large (corresponding to a higher global latency rate), then recombination must be rapidly integrating components of latent viruses into contemporary viruses. In general, the bottleneck strength and frequency were not very strongly identified, other than that extremely strong (i.e. perfect) and extremely frequent bottlenecks were ruled out for all patients (Figure S6), which is consistent with our observation that recombination alone even in the absence of bottleneck events can produce trees with the characteristic temporal ladder-like trees seen in within-host HIV phylogenies.

## Discussion

The importance of recombination and the latent reservoir in within-host HIV-1 evolutionary dynamics has been described in detail since the mid-1990s (Finzi et al. 1997; Ruelas and Greene 2013). Although these two processes have been modeled individually and together using forward mathematical models and simulations (Immonen et al. 2015; Doekes, Fraser, and Lythgoe 2017; Hill 2017; Murray et al. 2017), both processes have been largely ignored in phylogenetic and phylodynamic investigations because of the theoretical and computational problems in jointly inferring the phylogeny and recombination patterns. As efforts continue toward finding a functional cure of HIV-1 that involves eliminating or reducing the size of the latent reservoir, studying the interaction between the latent reservoir and recombination remains key. In this study, we extended a previously developed within-host coalescent model (Romero-Severson et al. 2014) into an ARG simulation model that allows for lineages to coalesce, recombine, and cycle in and out of a latent state. We found that these complex evolutionary dynamics leave tractable signals in bifurcating trees reconstructed using hierarchical clustering which can be quantified in readily measurable topological and distance statistics (Table 2).

We examined statistics computed from both maximum likelihood and minimum evolution empirical trees (Figure S4) finding that the statistics computed from the maximum likelihood trees were closer in alignment with what our simulator produced. Ultimately, it is unclear from a theoretical perspective how future simulation-based studies of recombination and phylogeny should proceed. However, our results in this context were very similar regardless of the choice of inference method for the empirical trees. Thus, our main result was that recombination restores the time structure in longitudinally sampled within-host HIV-1 phylogenies where HIV-1 has circled in and out of a latent reservoir. Hitherto, it was not understood why longitudinally sampled trees could show such a clear time structure (samples typically ordered in time along an unbalanced tree trunk), when recombination may mix everything, and latency makes old variants appear in more recent samples. Indeed, we show that without recombination, latency would have ruined the time structure. Taken together, however, recombination smooths out extremes and restores that disrupted time order. In fact, we show that recombination also smooths out effects of within-host bottlenecks.

Across the nine real patients analyzed, reassuringly, we found consistency between the recombination rates that produce the best-fitting trees to the empirical data and the current biological estimates of the effective recombination rate (1.4 × 10^−5^ to 1.38 × 10^−4^ per base per generation (Neher and Leitner 2010; Batorsky et al. 2011)), which corresponds to a rate of 0.008−0.081 recombination events per lineage per day in our simulation of a 700 nt genomic fragment). Interestingly, there was a positive correlation between the intensity of recombination and the global flow rate in and out of the latent reservoir. This leads to that large latent reservoirs will produce greater number of activation events, and consequently, faster recombination rates are required to maintain a ladder-like tree shape. While we found support for global flow rates in and out of the latent reservoir of 0.053 to 0.525 per day, corresponding to latent reservoirs of size 10^2^–10^3^, recall that our model assumes that the half-life of the latent reservoir is fixed at 44 months (Siliciano et al. 2003). Currently, however, it is not known what the size of the latent HIV reservoir is in a patient, and its dynamics over time are complicated as there exist different cell populations with different half-lives (White et al. 2022). It is therefore not possible to directly evaluate our latency results quantitatively. Also, because our model lacks a fitness cost for newly activated latent viruses, it is possible that we are inflating the recombination–latency correlation as reactivated latent cells that do not recombine with contemporary virus are not likely to survive long enough to be sampled at high frequency in reality (Immonen et al. 2015). Finally, similar to that the effective population size in a coalescent model does not match the census population size, our implied latent population size should also be thought of as an effective size. However, the relationship between latency dynamics and recombination in smoothing out the potential disruption of temporal ordering caused by latency is clear in the model. That is, in a neutral model, the size of the latent reservoir and the recombination rate must be correlated to obtain trees that look like typical within-host HIV phylogenies.

The third biological feature we included in our within-host ARG simulation model was the population demography in the form of (internal) bottlenecks. Changes in the population size can affect the accumulation of mutations, where transient reductions favor neutral drift over selection (Leitner and Kumar 2018). In our model, the coalescence rates of lineages are neutral between bottlenecks. However, low-level positive selection from the immune system would create fitness differences between lineages, especially for reactivated latent lineages that would be less fit because of long-term immunological memory (Richman et al. 2003; Wei et al. 2003; Bunnik et al. 2008). These fitness differences would likely lead to a more pronounced ladder-like structure of a tree and potentially improve the simulator by allowing for more variation in producing unbalanced trees (Figure S3). Thus, the lack of individual-level fitness differences between lineages in our model might explain the tendency of our model to produce somewhat more balanced trees than what may be seen in real HIV-1 trees. In addition to creating more balanced trees, this neutrality likely causes more variability in the possible evolutionary histories for a given set of parameters, particularly when recombination is low, potentially allowing for acceptance of low recombination parameter sets by chance alone.

In our distance algorithm, we introduced the relative distance matrix as a new statistical probe, which has two notable features. First, because the ARG models the time between events, the distance matrix obtained from an ARG-decomposed bifurcating tree accounts for the true evolutionary distance in time between the extant tips. Second, distance matrices can be computed from sequence data directly without first inferring a phylogenetic tree that may impose artifactual structures on the data in the context of high levels of recombination. However, a potential source of error comes from matching the time scale of the ARG to the genetic distance of the sequence data. In our formulation, an optimized scaling factor transforms the time distance to match the genetic distance and is independent of any specific genome region. The scaling factor assumes a constant rate over the course of an individual’s infection and assumes that multiple mutations at an individual site have been perfectly accounted for.

While our model advances the biological realism of within-host HIV-1 evolution, it could be further modified to include population structure. Population structure in terms of tissue compartmentalization may be an important feature that could reduce the range of possible evolutionary histories by limiting the lineages that can coalesce and recombine. At low, but non-zero migration rates, this could increase the number of lineages that survive through time by re-introducing un-recombined lineages into the target compartment and thus affect the ladder-like structure of simultaneously evolving lineages (like in P5 in Fig. 6B).

In conclusion, we have developed a novel HIV-1 within-host simulator that includes realistic features such as recombination, latency, and internal bottlenecks. These processes generate an ARG, a complex evolutionary structure formed by loops, mixed time signals, and unbalanced growth. Because such structures are, at this time, exceedingly difficult to infer from real data, we also developed an ARG decomposition method to visualize this complex structure as a familiar bifurcating tree. Together, these tools make it possible to match recombination, latency, and bottleneck effects to real HIV-1 within-host data, where the combined effects otherwise could not be analyzed.

## Supplementary Material

vead032_Supp

## Data Availability

The coalescent ARG simulator and ARG decomposition method are available at https://github.com/MolEvolEpid/ARG_simul-decomp.

## References

[R1] Batorsky R. et al. (2011) ‘Estimate of Effective Recombination Rate and Average Selection Coefficient for HIV in Chronic Infection’, *Proceedings of the National Academy of Sciences of the United States of America*, 108: 5661–6. 21436045 10.1073/pnas.1102036108 PMC3078368

[R2] Bunnik E. M. et al. (2008) ‘Autologous Neutralizing Humoral Immunity and Evolution of the Viral Envelope in the Course of Subtype B Human Immunodeficiency Virus Type 1 Infection’, *Journal of Virology*, 82: 7932–41. 18524815 10.1128/JVI.00757-08 PMC2519599

[R3] Chun T. W. et al. (1997) ‘Presence of an Inducible HIV-1 Latent Reservoir during Highly Active Antiretroviral Therapy’, *Proceedings of the National Academy of Sciences of the United States of America*, 94: 13193–7. 9371822 10.1073/pnas.94.24.13193 PMC24285

[R4] Crow J. F. , and Kimura M. (1965) ‘Evolution in Sexual and Asexual Population’, *The American Naturalist*, 99: 439–50.

[R5] Csardi G , and Nepusz T. (2006) The igraph software package for complex network research *InterJournal*, Complex Systems, 1695.

[R6] Desper R. , and Gascuel O. (2002) *Fast and Accurate Phylogeny Reconstruction Algorithms Based on the Minimum-Evolution Principle*. pp. 357–74. Berlin, Heidelberg: Springer. 10.1089/106652702761034136 12487758

[R7] Doekes H. M. , Fraser C., and Lythgoe K. A. (2017) ‘Effect of the Latent Reservoir on the Evolution of HIV at the Within- and Between-Host Levels’, *PLoS Computational Biology*, 13: e1005228. 10.1371/journal.pcbi.1005228 PMC5245781 28103248

[R8] Felsenstein J. (1974) ‘The Evolutionary Advantage of Recombination’, *Genetics*, 78: 737–56. 4448362 10.1093/genetics/78.2.737 PMC1213231

[R9] Finzi D. et al. (1997) ‘Identification of a Reservoir for HIV-1 in Patients on Highly Active Antiretroviral Therapy’, *Science (80-)*, 278: 1295–300. 10.1126/science.278.5341.1295 9360927

[R10] Fisher R. (1930) *The General Theory of Natural Selection*. Oxford: Clarendon.

[R11] Fisher M. et al. (2010) ‘Determinants of HIV-1 Transmission in Men Who Have Sex with Men: A Combined Clinical, Epidemiological and Phylogenetic Approach’, *AIDS*, 24: 1739–47. 20588173 10.1097/QAD.0b013e32833ac9e6

[R12] Gao F. et al. (2004) ‘Unselected Mutations in the Human Immunodeficiency Virus Type 1 Genome are Mostly Nonsynonymous and Often Deleterious’, *Journal of Virology*, 78: 2426–33. 14963138 10.1128/JVI.78.5.2426-2433.2004 PMC369203

[R13] Giardina F. et al. (2017) ‘Inference of Transmission Network Structure from HIV Phylogenetic Trees’, *PLoS Computational Biology*, 13: e1005316. 10.1371/journal.pcbi.1005316 PMC5279806 28085876

[R14] Giorgi E. E. et al. (2010) ‘Estimating Time since Infection in Early Homogeneous Hiv-1 Samples Using a Poisson Model’, *BMC Bioinformatics*, 11: 1–7. 20973976 10.1186/1471-2105-11-532 PMC2975664

[R15] Grabowski M. K. et al. (2014) ‘The Role of Viral Introductions in Sustaining Community-based HIV Epidemics in Rural Uganda: Evidence from Spatial Clustering, Phylogenetics, and Egocentric Transmission Models’, *PLoS Medicine*, 11: e1001610. 10.1371/journal.pmed.1001610 PMC3942316 24595023

[R16] Griffiths R. , and Marjoram P. (1996) ‘Ancestral Inference from Samples of DNA Sequences with Recombination’, *Journal of Computational Biology*, 3: 479–502. 9018600 10.1089/cmb.1996.3.479

[R17] Gu Z. et al. (1995) ‘Possible Involvement of Cell Fusion and Viral Recombination in Generation of Human Immunodeficiency Virus Variants That Display Dual Resistance to AZT and 3TC’, *The Journal of General Virology*, 76: 2601–5. 7595365 10.1099/0022-1317-76-10-2601

[R18] Guindon S. et al. (2005) ‘Phyml Online—a Web Server for Fast Maximum Likelihood-based Phylogenetic Inference’, *Nucleic Acids Research*, 33: W557–W559. 15980534 10.1093/nar/gki352 PMC1160113

[R19] Hall M. D. , and Colijn C. (2019) ‘Transmission Trees on a Known Pathogen Phylogeny: Enumeration and Sampling’, *Molecular Biology and Evolution*, 36: 1333–43. 30873529 10.1093/molbev/msz058 PMC6526902

[R20] Hein J. (1990) ‘Reconstructing Evolution of Sequences Subject to Recombination Using Parsimony’, *Mathematical Biosciences*, 98: 185–200. 2134501 10.1016/0025-5564(90)90123-g

[R21] Hill A. L. (2018) ‘Mathematical Models of HIV Latency’, *Current Topics in Microbiology and Immunology*, 417: 131–156. 29164341 10.1007/82_2017_77 PMC6117215

[R22] Ho Y.-C. et al. (2013) ‘Replication-Competent Noninduced Proviruses in the Latent Reservoir Increase Barrier to HIV-1 Cure’, *Cell*, 155: 540–51. 24243014 10.1016/j.cell.2013.09.020 PMC3896327

[R23] Immonen T. T. et al. (2015) ‘Recombination Enhances HIV-1 Envelope Diversity by Facilitating the Survival of Latent Genomic Fragments in the Plasma Virus Population’, *PLoS Computational Biology*, 11: e1004625. 10.1371/journal.pcbi.1004625 PMC4687844 26693708

[R24] Immonen T. T. , and Leitner T. (2014) ‘Reduced Evolutionary Rates in HIV-1 Reveal Extensive Latency Periods among Replicating Lineages’, *Retrovirology*, 11: 1–11. 25318357 10.1186/s12977-014-0081-0 PMC4201670

[R25] Janzen T. , Höhna S., and Etienne R. S. (2015) ‘Approximate Bayesian Computation of Diversification Rates from Molecular Phylogenies: Introducing a New Efficient Summary Statistic, the nLTT’, *Methods in Ecology and Evolution*, 6: 566–75.

[R26] Jetzt A. E. et al. (2000) ‘High Rate of Recombination Throughout the Human Immunodeficiency Virus Type 1 Genome’, *Journal of Virology*, 74: 1234–40. 10627533 10.1128/jvi.74.3.1234-1240.2000 PMC111457

[R27] Josefsson L. et al. (2013) ‘Single Cell Analysis of Lymph Node Tissue from HIV-1 Infected Patients Reveals That the Majority of CD4+ T-cells Contain One HIV-1 DNA Molecule’, *PLoS Pathogens*, 9: e1003432. 10.1371/journal.ppat.1003432 PMC3688524 23818847

[R28] Katoh K. , and Standley D. M. (2013) ‘MAFFT Multiple Sequence Alignment Software Version 7: Improvements in Performance and Usability’, *Molecular Biology and Evolution*, 30: 772–80. 23329690 10.1093/molbev/mst010 PMC3603318

[R29] Kellam P. , and Larder B. A. (1995) ‘Retroviral Recombination Can Lead to Linkage of Reverse Transcriptase Mutations That Confer Increased Zidovudine Resistance’, *Journal of Virology*, 69: 669–74. 7529334 10.1128/jvi.69.2.669-674.1995 PMC188627

[R30] Kondrashov F. A. , and Kondrashov A. S. (2001) ‘Multidimensional Epistasis and the Disadvantage of Sex’, *Proceedings of the National Academy of Sciences of the United States of America*, 98: 12089–92. 11593020 10.1073/pnas.211214298 PMC59772

[R31] Kouyos R. et al. (2010) ‘Molecular Epidemiology Reveals Long-term Changes in HIV Type 1 Subtype B Transmission in Switzerland’, *The Journal of Infectious Diseases*, 201: 1488–97. 20384495 10.1086/651951

[R32] Lee H. Y. et al. (2008) ‘Dynamic Correlation between Intrahost HIV-1 Quasispecies Evolution and Disease Progression’, *PLoS Computational Biology*, 4: e1000240. 10.1371/journal.pcbi.1000240 PMC2602878 19079613

[R33] Leitner T. (2019) ‘Phylogenetics in HIV Transmission: Taking Within-host Diversity into Account’, *Current Opinion in HIV and AIDS*, 14: 181–7. 30920395 10.1097/COH.0000000000000536 PMC6449181

[R34] Leitner T. , and Kumar S. (2018) ‘The Puzzle of HIV Neutral and Selective Evolution’, *Molecular Biology and Evolution*, 35: 1355–8. 29718409 10.1093/molbev/msy089 PMC6658841

[R35] Leitner T. , and Romero-Severson E. (2018) ‘Phylogenetic Patterns Recover Known HIV Epidemiological Relationships and Reveal Common Transmission of Multiple Variants’, *Nature Microbiology*, 3: 983–8. 10.1038/s41564-018-0204-9 PMC6442454 30061758

[R36] Mansky L. M. , and Temin H. M. (1995) ‘Lower in Vivo Mutation Rate of Human Immunodeficiency Virus Type 1 Than That Predicted from the Fidelity of Purified Reverse Transcriptase’, *Journal of Virology*, 69: 5087–94. 7541846 10.1128/jvi.69.8.5087-5094.1995 PMC189326

[R37] Mendes F. K. , Livera A. P., and Hahn M. W. (2019) ‘The Perils of Intralocus Recombination for Inferences of Molecular Convergence’, *Philosophical Transactions of the Royal Society B*, 374: 20180244. 10.1098/rstb.2018.0244 PMC6560264 31154973

[R38] Michod R. E. , Bernstein H., and Nedelcu A. M. (2008) ‘Adaptive Value of Sex in Microbial Pathogens’, *Journal of Molecular Epidemiology and Evolutionary Genetics in Infectious Diseases*, 8: 267–85. 18295550 10.1016/j.meegid.2008.01.002

[R39] Moradigaravand D. et al. (2014) ‘Recombination Accelerates Adaptation on a Large-scale Empirical Fitness Landscape in HIV-1’, *PLoS Genetics*, 10: e1004439. 10.1371/journal.pgen.1004439 PMC4072600 24967626

[R40] Moutouh L. , Corbeil J., and Richman D. D. (1996) ‘Recombination Leads to the Rapid Emergence of HIV1 Dually Resistant Mutants under Selective Drug Pressure’, *Proceedings of the National Academy of Sciences of the United States of America*, 93: 6106–11. 8650227 10.1073/pnas.93.12.6106 PMC39197

[R41] Muller H. J. (1932) ‘Some Genetic Aspects of Sex’, *The American Naturalist*, 66: 118–38.

[R42] Murray J. M. et al. (2017) ‘HIV Dynamics Linked to Memory CD4+ T Cell Homeostasis’, *PLoS One*, 12: e0186101. 10.1371/journal.pone.0186101 PMC5648138 29049331

[R43] Neher R. A. , and Leitner T. (2010) ‘Recombination Rate and Selection Strength in HIV Intra-patient Evolution’, *PLoS Computational Biology*, 6: e1000660. 10.1371/journal.pcbi.1000660 PMC2813257 20126527

[R44] Rasmussen M. D. et al. (2014) ‘Genome-Wide Inference of Ancestral Recombination Graphs’, *PLoS Genetics*, 10: e1004342. 10.1371/journal.pgen.1004342 PMC4022496 24831947

[R45] Rasmussen D. A. , Volz E. M., and Koelle K. (2014) ‘Phylodynamic Inference for Structured Epidemiological Models’, *PLoS Computational Biology*, 10: e1003570. 10.1371/journal.pcbi.1003570 PMC3990497 24743590

[R46] Ratmann O. et al. (2016) ‘Sources of HIV Infection among Men Having Sex with Men and Implications for Prevention’, *Science Translational Medicine*, 8: 320ra2. 10.1126/scitranslmed.aad1863 PMC4902123 26738795

[R47] Richman D. D. et al. (2003) ‘Rapid Evolution of the Neutralizing Antibody Response to HIV Type 1 Infection’, *Proceedings of the National Academy of Sciences of the United States of America*, 100: 4144–9. 12644702 10.1073/pnas.0630530100 PMC153062

[R48] Romero-Severson E. et al. (2014) ‘Timing and Order of Transmission Events Is Not Directly Reflected in a Pathogen Phylogeny’, *Molecular Biology and Evolution*, 31: 2472–82. 24874208 10.1093/molbev/msu179 PMC4137707

[R49] Romero-Severson E. O. , Bulla I., and Leitner T. (2016) ‘Phylogenetically Resolving Epidemiologic Linkage’, *Proceedings of the National Academy of Sciences of the United States of America*, 113: 2690–5. 26903617 10.1073/pnas.1522930113 PMC4791024

[R50] Romero-Severson E. , Meadors G., and Volz E. (2014) ‘A Generating Function Approach to HIV Transmission with Dynamic Contact Rates’, *Mathematical Modelling of Natural Phenomena*, 9: 121–35. 27087760 10.1051/mmnp/20149208 PMC4831738

[R51] Ruelas D. S. , and Greene W. C. (2013) ‘An Integrated Overview of HIV-1 Latency’, *Cell*, 155: 519–29. 24243012 10.1016/j.cell.2013.09.044 PMC4361081

[R52] Salminen M. O. et al. (1995) ‘Identification of Breakpoints in Intergenotypic Recombinants of HIV Type 1 by Bootscanning’, *AIDS Research and Human Retroviruses*, 11: 1423–5. 8573403 10.1089/aid.1995.11.1423

[R53] Shankarappa R. et al. (1999) ‘Consistent Viral Evolutionary Changes Associated with the Progression of Human Immunodeficiency Virus Type 1 Infection Downloaded From’, *Technical Report*, 12: 10489–502. 10.1128/jvi.73.12.10489-10502.1999 PMC113104 10559367

[R54] Siepel A. C. et al. (1995) ‘A Computer Program Designed to Screen Rapidly for HIV Type 1 Intersubtype Recombinant Sequences’, *AIDS Research and Human Retroviruses*, 11: 1413–6. 8573400 10.1089/aid.1995.11.1413

[R55] Siliciano J. D. et al. (2003) ‘Long-term Follow-up Studies Confirm the Stability of the Latent Reservoir for HIV-1 in Resting CD4+T Cells’, *Nature Medicine*, 9: 727–8. 10.1038/nm880 12754504

[R56] Skar H. et al. (2011) ‘Daily Sampling of an HIV-1 Patient with Slowly Progressing Disease Displays Persistence of Multiple Env Subpopulations Consistent with Neutrality’, *PLoS One*, 6: e21747. 10.1371/journal.pone.0021747 PMC3149046 21829600

[R57] Song H. et al. (2018) ‘Tracking HIV-1 Recombination to Resolve Its Contribution to HIV-1 Evolution in Natural Infection’, *Nature Communications*, 9: 1–15. 10.1038/s41467-018-04217-5 PMC5954121 29765018

[R58] Stadler T. et al. (2013) ‘Birth-death Skyline Plot Reveals Temporal Changes of Epidemic Spread in HIV and Hepatitis C Virus (HCV)’, *Proceedings of the National Academy of Sciences of the United States of America*, 110: 228–33. 23248286 10.1073/pnas.1207965110 PMC3538216

[R59] Volz E. M. et al. (2013) ‘HIV-1 Transmission during Early Infection in Men Who Have Sex with Men: A Phylodynamic Analysis’, *PLoS Medicine*, 10: 1–12. 10.1371/journal.pmed.1001568 PMC3858227 24339751

[R60] Volz E. M. , Romero-Severson E., and Leitner T. (2017) ‘Phylodynamic Inference across Epidemic Scales’, *Molecular Biology and Evolution*, 34: 1276–88. 28204593 10.1093/molbev/msx077 PMC5400386

[R61] Wakeley J. (2009) *Coalescent Theory: An Introduction*. Greenwood Village, Colo: Roberts & Co. Publishers.

[R62] Wang L. , Zhang K., and Zhang L. (2001) ‘Perfect Phylogenetic Networks with Recombination’, *Journal of Computational Biology*, 8: 69–78. 11339907 10.1089/106652701300099119

[R63] Wei X. et al. (2003) ‘Antibody Neutralization and Escape by HIV-1’, *Nature*, 422: 307–12. 12646921 10.1038/nature01470

[R64] White J. A. et al. (2022) ‘Complex Decay Dynamics of HIV Virions, Intact and Defective Proviruses, and 2ltr Circles following Initiation of Antiretroviral Therapy’, *Proceedings of the National Academy of Sciences*, 119: e2120326119. 10.1073/pnas.2120326119 PMC8833145 35110411

[R65] Wong J. K. et al. (1997) ‘Recovery of Replication-Competent HIV Despite Prolonged Suppression of Plasma Viremia’, *Science (80-)*, 278: 1291–5. 10.1126/science.278.5341.1291 9360926

[R66] Zanini F. et al. (2015) ‘Population Genomics of Intrapatient HIV-1 Evolution’, *eLife*, 4: e11282. 10.7554/eLife.11282 PMC4718817 26652000

[R67] Zhuang J. et al. (2002) ‘Human Immunodeficiency Virus Type 1 Recombination: Rate, Fidelity, and Putative Hot Spots’, *Journal of Virology*, 76: 11273–82. 12388687 10.1128/JVI.76.22.11273-11282.2002 PMC136766

