# Supplementary material for: Recombination smooths the time signal disrupted by latency in within-host HIV phylogenies
Source: Virus Evol. 2023 May 20;9(1):vead032. doi: 10.1093/ve/vead032 (PMC10313349; doi:10.1093/ve/vead032)
Supplement: vead032_Supp [file vead032_supp.zip › suppl_data/Castro_recombination_supplement.docx]

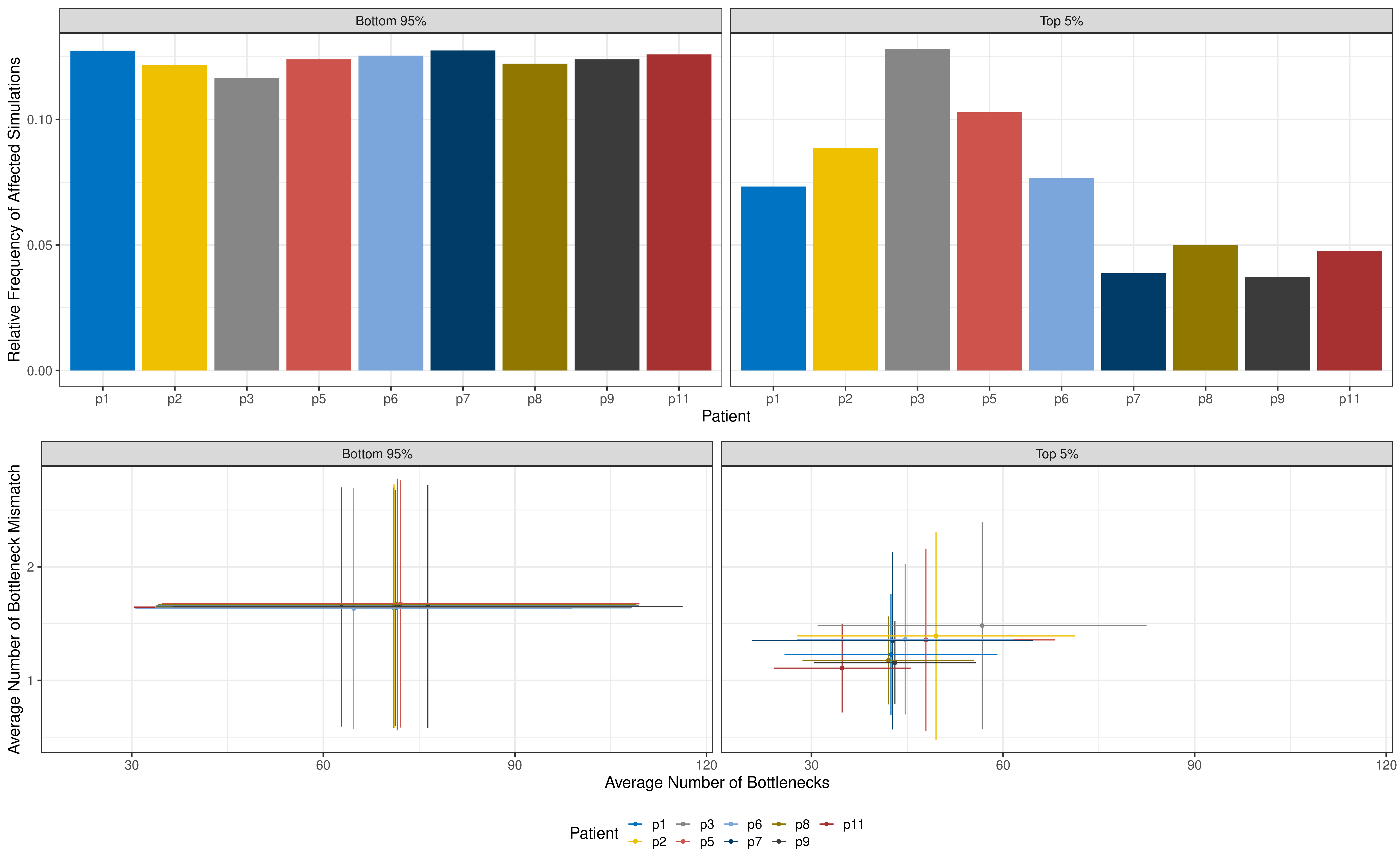


Figure S1: Analysis of bottleneck events where $B_{\text{strength}} > N(t)$. (A) The relative frequency of simulations in which there is at least one instance where $B_{\text{strength}} > N(t)$. The right column represents simulations that are in the top 5% of matching simulations. (B) The average number of bottlenecks in a simulation against the average number of bottleneck incongruities. Error bars presents one standard deviation in each direction. For all patients, the average number of bottleneck incongruities is less than 2 and represents 3% of bottlenecks within a simulation.


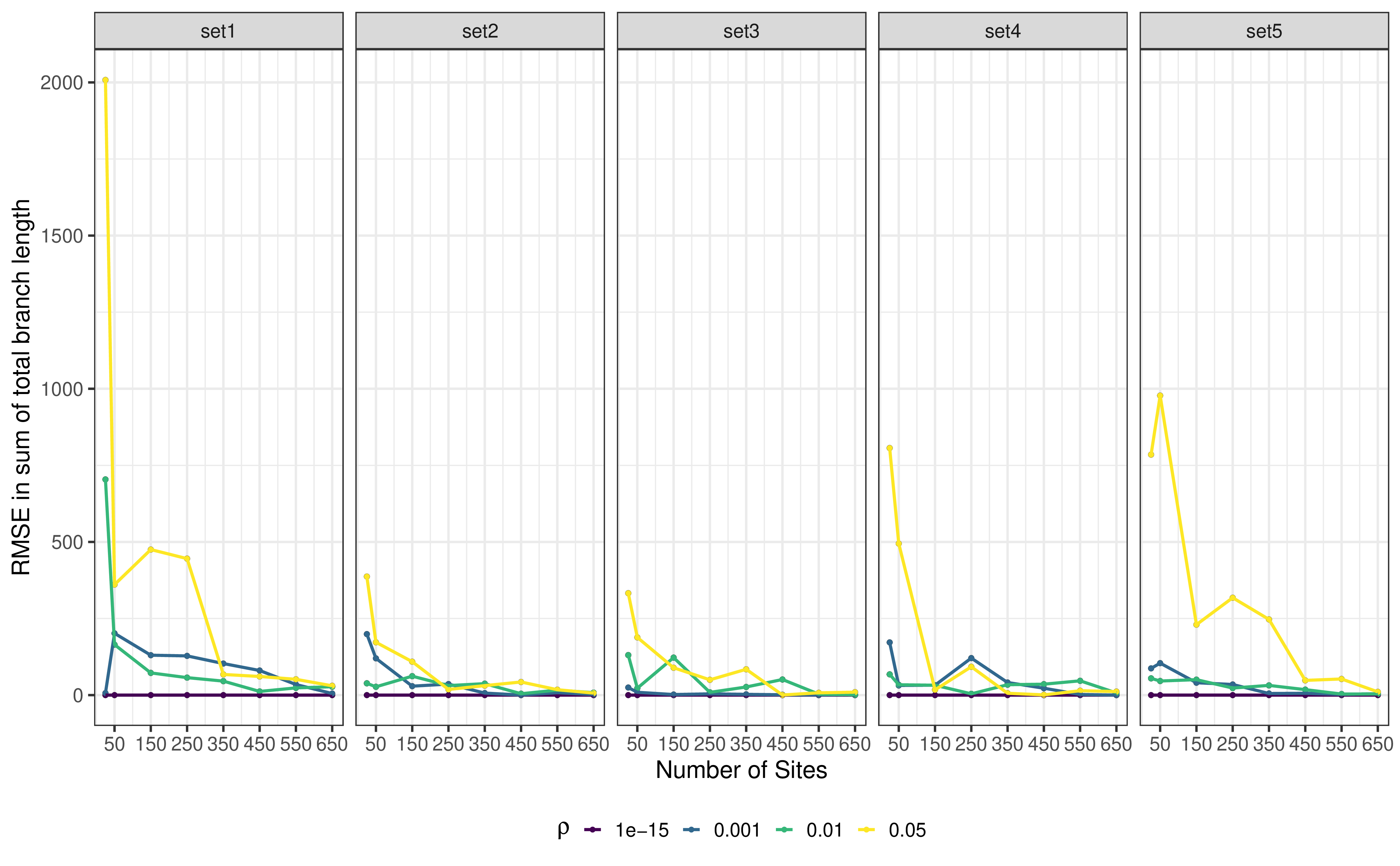


Figure S2: Sensitivity of reconstructed tree to number of decomposed sites. The root mean squared error (RMSE) in sum of the total branch length measures the distance between a reconstructed tree using a subset of the 700 residues and the reconstructed tree using all 700 residues. Colors represent different values of the recombination rate ρ. We did not explore ρ = 0.1 for computational efficiency but observe the trend that the reconstructed tree is more sensitive to the number of sampling sites as the recombination rate increases. Each column represents a random sample of the other three parameters (latent reservoir site, $B_{\text{strength}}$ and $B_{\text{frequency}}$).


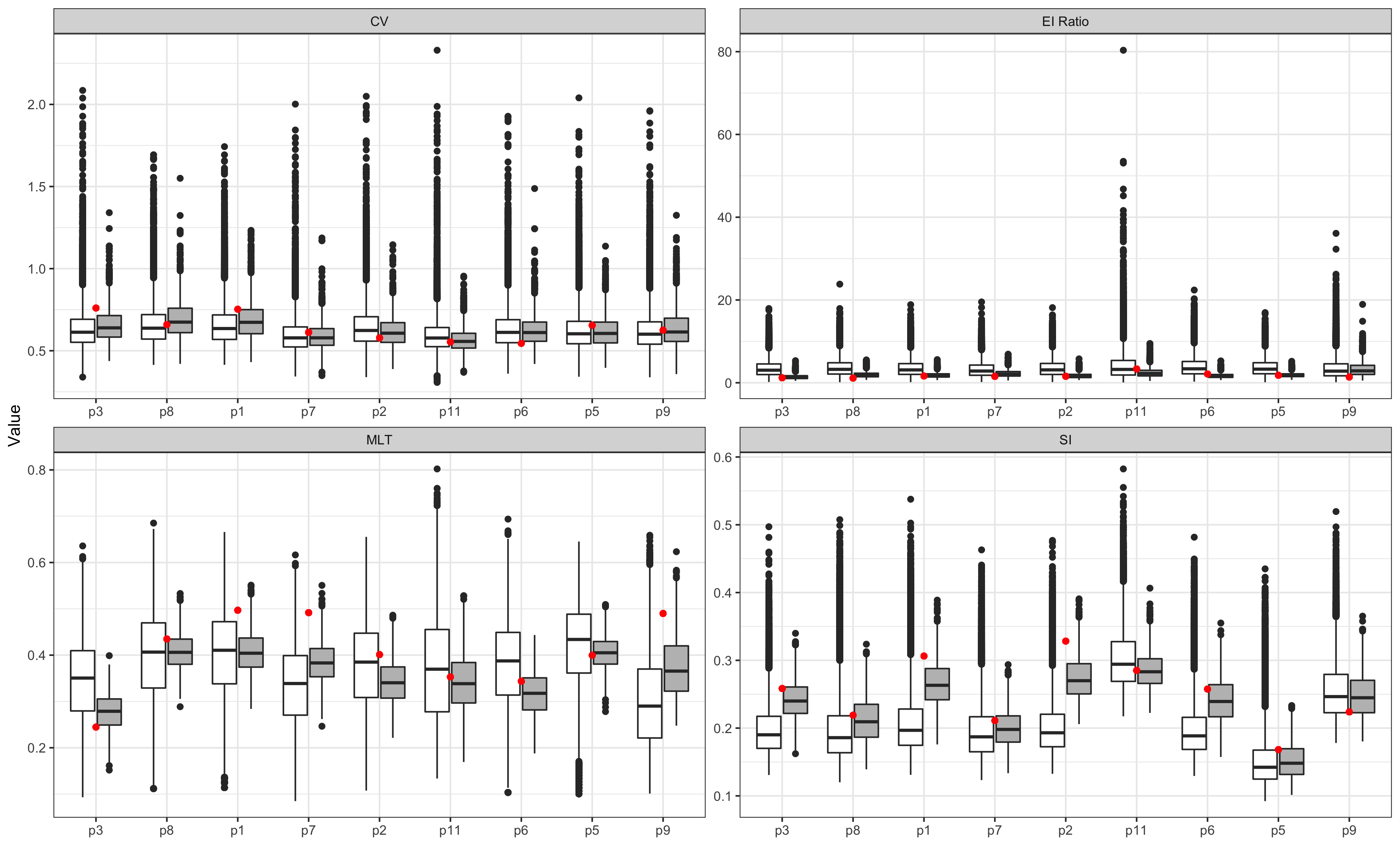
Figure S3: Distributions of topological and distance statistics by top 5% (grey) and bottom 95% (black) of the scored ARG-simulated reconstructed phylogenies for each patient. The extents of the box and correspond to the interquartile range; the bars extend to 1.5 of the IQR. Outliers are shown. The red dot marks the empirical value. Patients are ordered according to the normalized score, from best-fitting to worst-fitting.


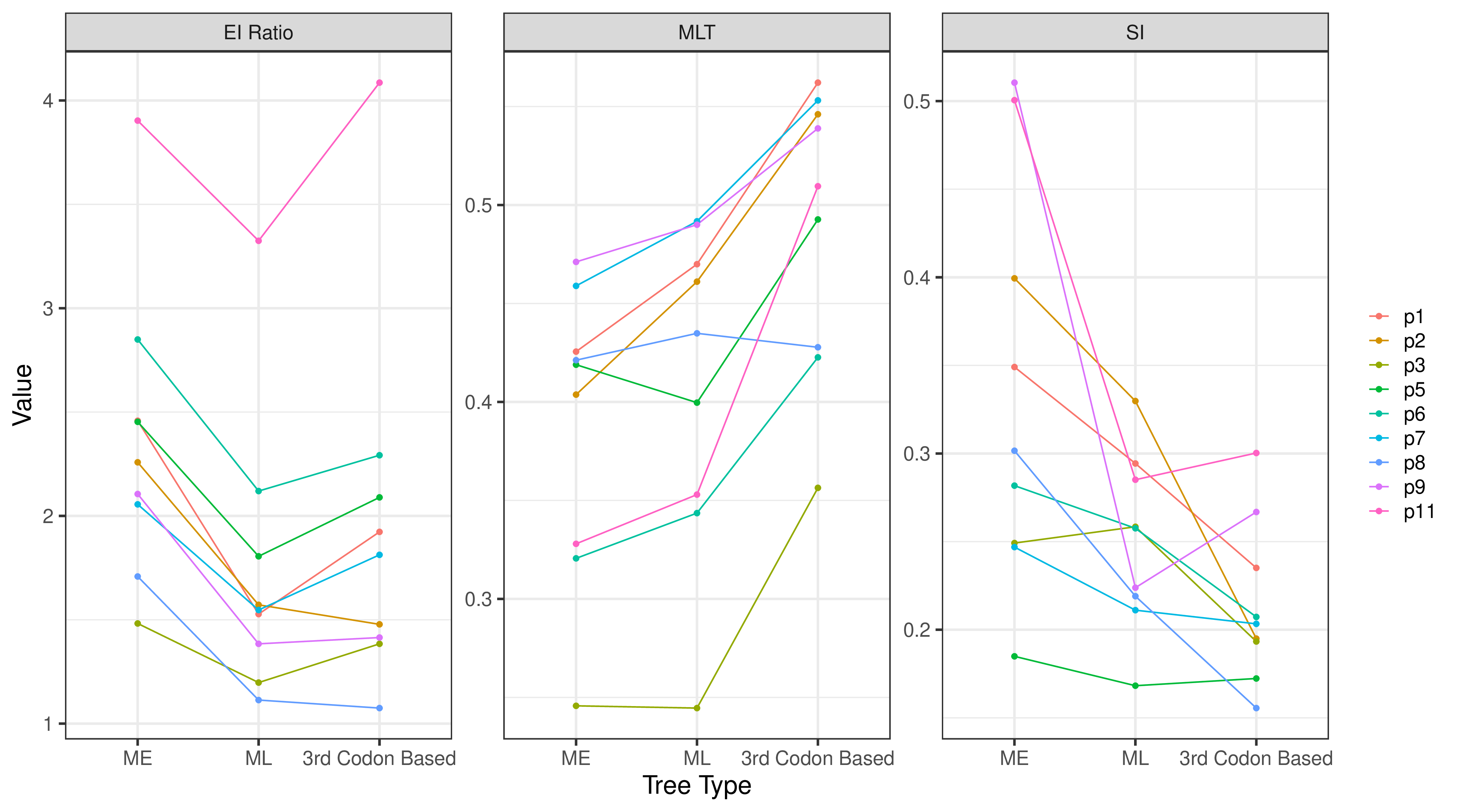
Figure S4: Comparison of topological tree raw statistics across three tree reconstruction methods ME refers to trees reconstructed using the Maximum Evolution algorithm and those used in the main analysis; ML are trees reconstructed using Maximum Likelihood; 3rd codon based trees are those computed on the 3rd codon position only. All trees were rooted by tMRCA of the first time point and limited to the first 90 months of samples. Statistics across an individual patient are connected by a line and color.


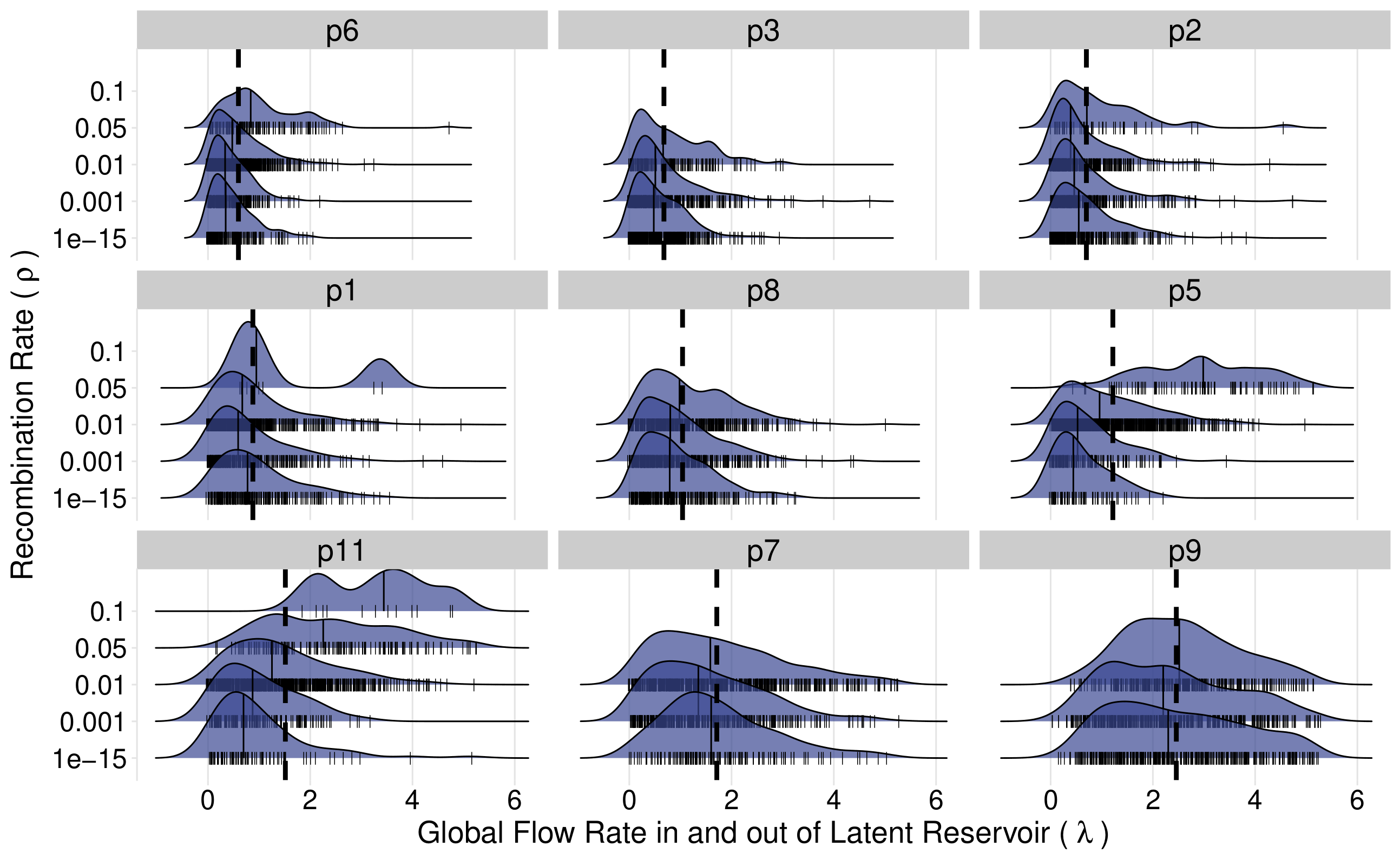
Figure S5: Distribution of global flow rate in and out of latent reservoir stratified by recombination rate. For each patient, the posterior samples for reservoir size are shown stratified by recombination rate. The dashed line shows the maximum posterior estimated of the reservoir size for each patient. Patients are ordered by increasing mean of $\lambda$ of the best-fitting 5% of simulations.


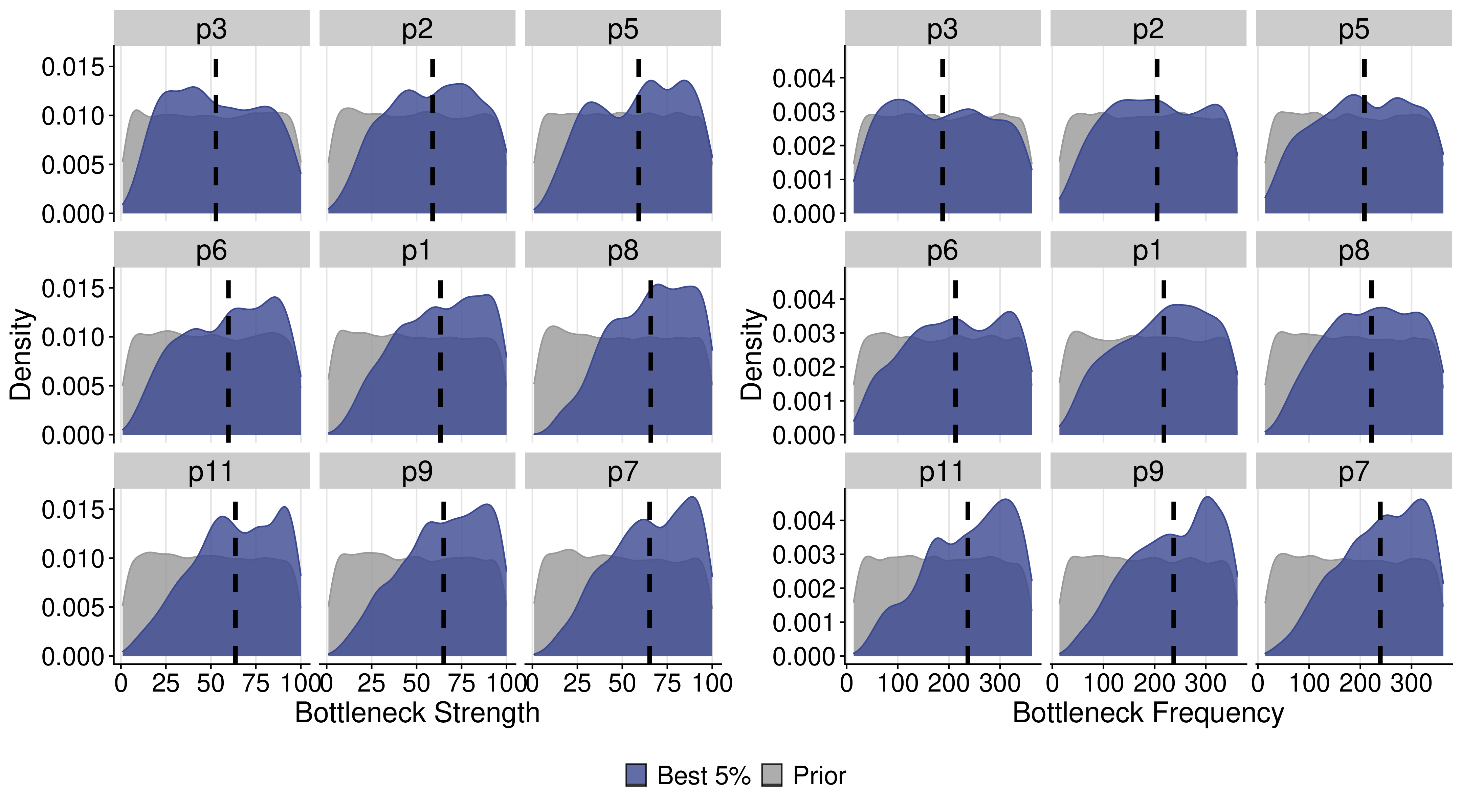


Figure S6: Marginal distributions of the bottleneck attributes of strength and frequency across the nine patients. Colors distinguish the density of parameters between simulations with the best (lowest) 5% of distance scores (red) and the highest 95% of distance scores (blue). The dashed line indicates the mean of the best-fitting 5% of simulations. Patients are ordered by the best-fitting 5% mean of bottleneck frequency values.
